# Investigating depression pathways in a clinical sample: childhood adversity, reward, and threat processing

**DOI:** 10.3389/fpsyg.2025.1448585

**Published:** 2025-10-09

**Authors:** Lia-Ecaterina Oltean, Andrei C. Miu, Aurora Szentágotai-Tǎtar

**Affiliations:** ^1^The International Institute for the Advanced Studies of Psychotherapy and Applied Mental Health, Babeş-Bolyai University, Cluj-Napoca, Romania; ^2^Department of Clinical Psychology and Psychotherapy, Babeş-Bolyai University, Cluj-Napoca, Romania; ^3^Cognitive Neuroscience Laboratory, Department of Psychology, Babeş-Bolyai University, Cluj-Napoca, Romania

**Keywords:** childhood adversity, depression, socioeconomic status, reward, threat

## Abstract

Childhood adversity (CA, abuse and neglect) is an important risk factor for depression. However, other variables, such as childhood socioeconomic status (SES), may influence this association. Moreover, the underlying mechanisms that contribute to the devastating consequences of CA for depression are incompletely understood. Still, recent work point toward reward and threat processing. Building on these, we sought (1) to investigate associations between CA, depression, reward and threat processing, (2) to control for childhood SES when investigating these associations, and (3) to test the mediating role of reward and threat processing in the association between CA and depression. Inpatients (*N* = 33, *m* = 48.36; *SD* = 11.76) with a formal diagnosis of major depressive disorder completed self-report measures for all variables of interest. We found significant associations between CA, depression and threat processing, but no other significant associations emerged (*p* < 0.05). Results remained similar when we controlled for childhood SES. Furthermore, we did not find support for the potential mediating role of reward and threat processing in the association between CA and depression. These results extend existing research investigating CA and depression, and single out threat processing. Moreover, they suggest that these patterns of associations are robust. While they do not provide support for the proposed mechanisms, they underscore the need for further research and point out relevant directions.

## Introduction

Depression is characterized by persistent sadness and lack of interest and pleasure (American Psychiatric Association, 2013). These symptoms are accompanied by various physical, emotional, cognitive and behavioral changes, such as appetite or sleep, excessive guilt, suicidal thoughts or behavior (American Psychiatric Association, 2013). In the last decades, these symptoms have been increasing continuously, making depression one of the most common mental conditions (Liu et al., 2020). In turn, this has led to tremendous social and individual costs (Biesheuvel-Leliefeld et al., 2016). These costs have been further exacerbated by symptom severity (Richards, 2011). Notably, greater impairment in depression is associated with both increased help-seeking and poor treatment response (Bromet et al., 2018; De Carlo et al., 2016). Therefore, the scientific community has been keen on investigating potential risk factors implicated in its etiology.

One of the most important risk factors for depression is childhood adversity (i.e., CA; e.g., Humphreys et al., 2020). Supporting this view, CA predicts symptom onset, illness course, and severity of depression (Hovens et al., 2012). Furthermore, it may influence treatment response, suggesting that individuals with CA are more difficult to treat (Nanni et al., 2012). However, several aspects hamper our understanding on CA pathways to depression and may require further clarification.

First, it has been argued other variables are potential confounds that may influence the association between CA and depression. Among these, research points toward childhood socioeconomic status (i.e., SES). Of relevance, CA often occurs in low SES communities (McLaughlin et al., 2011). Moreover, childhood SES predicts depression (Morrissey and Kinderman, 2020). Thus, acknowledging its potential influence in research on CA and depression holds promise to increase current understanding on this topic.

Second, underlying mechanisms for the association between CA and depression are largely unknown (Gerin et al., 2019). Whereas several promising mechanisms exist, emerging data favor reward processing (Harms et al., 2019; Gerin et al., 2019). Consistent with this view, data support CA’s implications for reward processing (e.g., for a review, see Oltean et al., 2023), and highlight reward processing in depression (for a review, see Halahakoon et al., 2020), Moreover, there is direct support for this mechanism (e.g., Oltean and Şoflǎu, 2022). Nevertheless, as this evidence is sparse and inconsistent, the complex functions of the reward processing system warrant attention.

This system is implicated in the acquisition, consumption and learning based on rewards (i.e., positive stimuli that motivate behavior), and it has three distinct dimensions: reward responsiveness, reward learning and reward valuation (National Advisory Mental Health Council Workgroup on Tasks and Measures for Research Domain Criteria, 2016). Hence, impairments may occur in any of them. Aligned with this view, associations between CA and reward processing vary by dimension (Oltean et al., 2023), and reward processing in depression follows similar patterns (Halahakoon et al., 2020). Furthermore, recent work suggests that among reward processing dimensions, reward learning mediates the association between CA and depression (e.g., Oltean and Şoflǎu, 2022). Still, given that support for this mechanism is limited, other mechanisms are plausible.

Threat processing, the ability to detect and respond to negative stimuli (i.e., punishment), is another potential mechanism (Gerin et al., 2019). Consistent with this view, threat processing impairments follow CA (Weissman et al., 2020). They are mostly characterized by hypervigilance toward threats and diminished ability to discriminate between threat and other affective cues (Gerin et al., 2019; Lange et al., 2019). Although investigated less (Craske, 2012), similar impairments have been documented in depression (e.g., Lange et al., 2019; Li et al., 2015; Sportel et al., 2011). Moreover, there is some evidence that threat processing mediates the association between CA and depression (Miu et al., 2017). Yet, mirroring evidence on reward processing, direct support for the mediating role of threat processing is scant and inconsistent (e.g., Gerin et al., 2024).

Building on these, we aimed (1) to investigate the associations between CA, depression, reward and threat processing. Moreover, we sought (2) to control for childhood SES when investigating these associations. We grounded our decision both on literature suggesting that childhood SES is a potential confound (McLaughlin et al., 2011), and work that documents its association with reward (Romens et al., 2015) and threat processing (Swartz et al., 2017). Finally, considering that identifying underlying mechanisms has crucial implications for treatment (Danese, 2020), we aimed (3) to test the potential mediating role of both reward and threat processing in the association between CA and depressive symptoms.

## Methods

### Sample

We recruited participants from a psychiatric unit located in Romania, by disseminating information to eligible inpatients. Inclusion criteria were age (being older than 18 years) and formal diagnosis of major depressive disorder (i.e., MDD). Given that MDD has high comorbidity rates with other mental (e.g., Wilson et al., 2020; Hunt et al., 2020) and physical conditions (e.g., Gold et al., 2020), we did not exclude participants if they presented with comorbidities. Exclusion criteria were age (i.e., being younger than 18 years) and the absence of a formal diagnosis of MDD. This procedure resulted in recruiting a sample of 33 participants (*m* = 48.36; *SD* = 11.76). Consistent with studies that document women are more likely to suffer from depression (e.g., up to 2:1 female:male ratio; Piccinelli and Wilkinson, 2000) and seek treatment (Parker and Brotchie, 2010), most participants were women (72.7%).

All participants provided an informed consent and completed the measures on site, while being hospitalized. All procedures contributing were aligned with the ethical standards of the relevant national and institutional committees on human experimentation and with the Helsinki Declaration of 1975, as revised in 2008. Only partial data was obtained for some measures and data collection was stopped due to COVID-19 restrictions in place.

### Measures

#### Sociodemographic variables

In order to reduce participant burden, we prioritized collecting data that were essential to the study objectives. As such, we collected sociodemographic data on age and gender. We constructed a socioeconomic status scale building on guidelines that recommend using indicators related to the educational, occupational, and poverty level (McLaughlin et al., 2011). The scale exhibited acceptable internal consistency (Cronbach’s α = 0.67).

#### Childhood adversity

We evaluated CA with The Childhood Trauma Questionnaire (CTQ; Bernstein et al., 1997). Such retrospective measures are particularly useful in research on psychopathology as they capture the subjective nature of CA (Baldwin et al., 2024). Indeed, this scale has been previously used in research investigating CA and depression (Humphreys et al., 2020). The CTQ evaluates experiences of abuse and neglect, yet these experiences usually co-occur (Smith and Pollak, 2021). Hence, we computed an overall CA score. The CTQ has good reliability and validity (Bernstein et al., 1997; Georgieva et al., 2021) and it displayed good internal consistency in this study (Cronbach’s α = 0.93).

#### Depression

While participants were formally diagnosed with MDD by a psychiatrist, we also evaluated symptom level depression with a self-report measure, the Patient Health Questionnaire-9 (PHQ-9; Kroenke et al., 2001). The PHQ-9 is a widely used measure (Negeri et al., 2021). It is brief and has been successfully used across different settings, including samples of clinically depressed inpatients (Costantini et al., 2021). The PHQ-9 exhibits good reliability and validity (Levis et al., 2019) and it had adequate internal consistency in our sample (Cronbach’s α = 0.64).

#### Reward processing

We assessed reward processing using the Behavioral Activation System Subscale of the Behavioral Inhibition System and Behavioral Activation System Scales (BIS/BAS; Carver and White, 1994). The BIS/BAS is a self-report measure, which has good reliability and validity across different samples (e.g., Jorm et al., 1998; Franken et al., 2005; Shi et al., 2025). It has been validated on the Romanian population (Sava and Sperneac, 2006), and has been previously used in similar research investigating reward processing (e.g., Mattoni et al., 2024; Oltean and Şoflǎu, 2022). In our sample, the BAS subscale exhibited good internal consistency (Cronbach’s α = 0.84).

Given that reward learning, responsiveness and valuation have been conceptualized as distinct dimensions of reward processing (National Advisory Mental Health Council Workgroup on Tasks and Measures for Research Domain Criteria, 2016), we also evaluated these constructs separately. We selected self-report measures based on existing guidelines (National Advisory Mental Health Council Workgroup on Tasks and Measures for Research Domain Criteria, 2016), and prior work examining CA, reward processing and depression (e.g., Oltean and Şoflǎu, 2022). As such, we evaluated *reward responsiveness* with the Reward Responsiveness subscale of the BIS/BAS (Carver and White, 1994). In our sample, this subscale displayed acceptable internal consistency in our study (Cronbach’s α = 0.65). Moreover, we assessed *reward learning* with The Reward Expectancy subscale of The Generalized Reward and Punishment Expectancy Scales (GRAPES; Ball and Zuckerman, 1990). In this study, this subscale displayed adequate internal consistency (Cronbach’s α = 0.79). Finally, we measured *reward valuation* with The Drive Subscale of the BIS/BAS (Carver and White, 1994). In this sample, the subscale exhibited acceptable internal consistency (Cronbach’s α = 0.66).

#### Threat processing

We evaluated threat processing using two distinct measures. Consistent with existing guidelines (National Advisory Mental Health Council Workgroup on Tasks and Measures for Research Domain Criteria, 2016) and prior work (e.g., Miu et al., 2017), we measured threat processing with the Behavioral Inhibition System subscale of the BIS/BAS (Carver and White, 1994). In addition, we used the Punishment Expectancy subscale of the GRAPES (Ball and Zuckerman, 1990). The BIS had moderate internal consistency in this study (Cronbach’s α = 0.51). Given that the Punishment Expectancy subscale did not exhibit acceptable internal consistency (Cronbach’s α = 0.35) we did not use it.

### Data analysis

All statistical analyses were performed using JASP 0.15 (JASP Team, 2021). We computed descriptive statistics for sociodemographic variables, and other relevant variables, and used Pearson correlations (*r*) to test associations between variables, as well as partial correlations in order to control for childhood SES. Given the ongoing debate and mixed empirical evidence on traditional and alternative bootstrapping methods for testing mediation (e.g., Igartua and Hayes, 2021; Leth-Steensen and Gallitto, 2016), we adopted a two-step approach when investigating the hypothesized mechanisms. First, we applied a joint significance test (Fritz et al., 2012; Kenny and Judd, 2014) by examining the correlation patterns to determine whether significant associations emerge between the hypothesized mediating variables and both the predictor (path a) and the outcome (path b). Second, we conducted mediation analyses using multiple regression with 5,000 bootstraps (Hayes, 2009), testing the models that met the conditions outlined in the joint significance test.

## Results

### Descriptive statistics

Table 1 indicates descriptive statistics for all variables. The mean score for depressive symptoms was above the cut-off score for severe depression (Kroenke et al., 2001), and the mean score for CA was above the 95th percentile (Scher et al., 2001).

**TABLE 1:** Descriptive statistics.

	Minimum	Maximum	Mean	SD
PHQ	10.00	26.00	20.58	3.79
CTQ	25.00	97.00	58.27	22.63
BAS	17.00	48.00	34.37	8.51
BAS-drive	5.00	16.00	10.57	3.09
BAS-rew	5.00	20.00	13.50	3.77
Grapes-rew	0.00	12.00	4.16	3.18
BIS	15.00	28.00	24.43	3.07
SES-ch	7.00	16.00	11.45	2.06
Age	26	79	48.36	11.76

CTQ, Childhood Trauma Questionnaire (Bernstein et al., 1997); PHQ, Patient Health Questionnaire (Kroenke et al., 2001); BAS, Behavioral Activation System subscale of The Behavioral Inhibition System and Behavioral Activation System Scales (Carver and White, 1994); BAS-drive, Reward Drive subscale of The Behavioral Inhibition System and Behavioral Activation System Scales (Carver and White, 1994); BAS-rew, Reward Responsiveness Subscale of The Behavioral Inhibition System and Behavioral Activation System Scales (Carver and White, 1994); Grapes-rew: Reward Expectancy subscale of The Generalized Reward and Punishment Expectancy Scales (Ball and Zuckerman, 1990); BIS, Behavioral Inhibition System subscale of The Behavioral Inhibition System and Behavioral Activation System Scales (Carver and White, 1994); SES-ch, Socioeconomic Status during childhood.

### Associations between variables

CA was positively associated with high severity depressive symptoms (see Table 2), but not with reward processing. Likewise, no associations between reward processing and depressive symptoms emerged. In contrast, both CA and depressive symptoms were significantly associated with threat processing (see Table 2).

**TABLE 2:** Correlation matrix.

	PHQ	BAS	BAS-drive	BAS-rew	Grapes-rew	BIS	SES-ch
CTQ	0.436*	0.156	0.061	0.095	–0.013	0.462*	0.065
PHQ	1	0.142	0.184	–0.106	0.033	0.412*	0.234
BAS		1	0.871**	0.881**	0.450*	0.081	0.300
BAS-drive			1	0.653**	0.434*	–0.027	0.298
BAS-rew				1	0.252	0.010	0.174
Grapes-rew					1	–0.099	0.629**
BIS						1	–0.112

**p < 0.01 (2-tailed), *p < 0.05 (2-tailed). CTQ, Childhood Trauma Questionnaire (Bernstein et al., 1997); PHQ, Patient Health Questionnaire (Kroenke et al., 2001); BAS, Behavioral Activation System subscale of The Behavioral Inhibition System and Behavioral Activation System Scales (Carver and White, 1994); BAS-drive, Reward Drive subscale of The Behavioral Inhibition System and Behavioral Activation System Scales (Carver and White, 1994); BAS-rew, Reward Responsiveness Subscale of The Behavioral Inhibition System and Behavioral Activation System Scales (Carver and White, 1994); Grapes-rew: REWARD Expectancy subscale of The Generalized Reward and Punishment Expectancy Scales (Ball and Zuckerman, 1990); BIS, Behavioral Inhibition System subscale of The Behavioral Inhibition System and Behavioral Activation System Scales (Carver and White, 1994); SES-ch, Socioeconomic Status during childhood.

After controlling for childhood SES, results followed a similar pattern (see Table 3).

**TABLE 3:** Partial correlations—controlling for childhood SES.

	PHQ	BAS	BAS-drive	BAS-rew	Grapes-rew	BIS
CTQ	0.396*	0.116	–0.008	0.043	–0.070	0.441*
PHQ	–	0.007	0.041	–0.252	–0.204	0.422*
BAS		–	0.854**	0.873**	0.310	0.069
BAS-drive			–	0.615**	0.272	–0.062
BAS-rew				–	0.117	–0.033
Grapes-rew					–	–0.105

**p < 0.01 (2-tailed), *p < 0.05 (2-tailed). CTQ, Childhood Trauma Questionnaire (Bernstein et al., 1997); PHQ, Patient Health Questionnaire (Kroenke et al., 2001); BAS, Behavioral Activation System subscale of The Behavioral Inhibition System and Behavioral Activation System Scales (Carver and White, 1994); BAS-drive, Reward Drive subscale of The Behavioral Inhibition System and Behavioral Activation System Scales (Carver and White, 1994); BAS-rew, Reward Responsiveness Subscale of The Behavioral Inhibition System and Behavioral Activation System Scales (Carver and White, 1994); Grapes-rew: Reward Expectancy subscale of The Generalized Reward and Punishment Expectancy Scales (Ball and Zuckerman, 1990); BIS, Behavioral Inhibition System subscale of The Behavioral Inhibition System and Behavioral Activation System Scales (Carver and White, 1994).

### Mediation analyses

As reward-processing measures were not significantly associated with CA and depressive symptoms, they did not meet conditions outlined in the joint significance test (Table 2). Thus, we did not run bootstrapping procedures for the potential mediating role of reward processing and found no evidence that it may partially explain the link between CA and depression.

The regression analysis testing the potential mediating role of threat processing in the association between CA and depressive symptoms indicated that there is a total effect of CA on depressive symptoms (*b* = 0.07, *SE* = 0.03, *CI* [0.02; 0.13]). We did not find a significant indirect effect (*b* = 0.02, *SE* = 0.01, *CI* [-0.02; 0.08]) and the direct effect remained significant (*b* = 0.06, *SE* = 0.03, *CI* [0.004; 0.12]).

## Discussion

This study investigated associations between CA, depression, reward and threat processing in a sample of clinically depressed inpatients. Seeking to clarify current understanding, the study investigated these associations while controlling for childhood SES, an important potential confound. Moreover, the study identified and investigated two potential mechanisms implicated in the association between CA and depression: reward and threat processing. Thus, its findings have several important contributions. We found that higher levels of CA were associated with increased symptom severity. While this finding is largely consistent with existing literature (e.g., Humphreys et al., 2020), those on reward processing apparently contrast it. Notably, we did not replicate prior findings that document CA’s implications for reward processing (Oltean et al., 2023), and reward processing in depression (e.g., Halahakoon et al., 2020). Though our results were aligned with studies that do not report significant associations between CA and reward processing (e.g., Marusak et al., 2017), and reward processing in depression (Li et al., 2015), this heterogenous literature may suggest additional research in this area is needed.

Moreover, we found threat processing was associated with CA, a finding that adds to prior ones documenting this association (Lange et al., 2019). In addition, we found a significant association between threat processing and depression, which expands on the arguably limited work in this area (Craske, 2012; Kasch et al., 2002, Medeiros et al., 2020). Together, our findings may suggest that both CA and depression may be coupled with an overly reactive threat processing system.

Furthermore, when we controlled for childhood SES, all significant associations remained stable, and no other associations emerged. This finding challenges prior views on this potential confound (Gerin et al., 2019), and should be interpreted with caution given that participants exhibited little variation on childhood SES. However, it may suggest that the observed pattern of associations between variables is robust and is not merely the result of childhood SES exerting an influence on them. While tentative, this view is aligned with data indicating that although childhood SES predicts depression, CA may largely explain this effect (Yang et al., 2021).

Prior data suggest reward processing is a potential mechanism implicated in the association between CA and depression (Harms et al., 2019, Gerin et al., 2024). Still, contrary to our expectations, we did not find support for this hypothesis. While surprising, methodological differences between studies may explain our findings. We evaluated reward processing using self-report measures. Although other studies employed a similar approach (e.g., Marusak et al., 2017; Oltean and Şoflǎu, 2022), most have relied on neural or cognitive tasks (e.g., Halahakoon et al., 2020). Given that distinct measures tap into different features of reward processing (e.g., Mattoni et al., 2024), further research is needed to draw more definitive conclusions regarding this potential mechanism.

Likewise, the mediating role of threat processing may require additional clarification, as some work support it (e.g., Miu et al., 2017), while others do not (e.g., Gerin et al., 2024; Lange et al., 2019). Even though our findings are consistent with the latter, one possible explanation to the conflicting results existing in literature pertains to distinct sample characteristics across various studies. While prior work has been mostly conducted on community samples, participants in this study were inpatients, with a formal diagnosis of MDD. Given that inpatients often exhibit specific clinical manifestations, including more severe symptoms and comorbidities, as well as poorer treatment responses (Bromet et al., 2018; Cheng et al., 2007), these differences may explain our results.

Despite its contributions, this study has several limitations that should be noted. The cross-sectional design used here does not warrant establishing temporal sequence or unidirectional links between variables. Hence, alternative models cannot be excluded. For example, we grounded our model on work that assumes reward processing impairments precede symptoms (e.g., Hanson et al., 2015). Given that these impairments may also follow depression (Nielson et al., 2021), future longitudinal studies exploring these pathways are needed. Moreover, recent work indicates mechanisms may interact with one another (e.g., Li et al., 2020). Still, our study focused on reward and threat processing separately, and did not investigate their interaction with one another, nor with other documented mechanisms (e.g., emotional regulation; Miu et al., 2022). Therefore, future studies investigating CA and depression may benefit from an in-depth examination of both the interactions between reward and threat processing, and their interplay with other mechanisms.

Furthermore, this study was conducted on a small sample and may lack statistical power needed to detect smaller significant associations. In addition, participants were mostly women, with poor childhood SES, which may contribute to a flooring effect and limit generalizing results. We suggest future research using larger samples and more diverse populations may counter these limitations. Last, exclusive reliance on self-report measures may limit the interpretation of our results. Subjective measures of reward and threat processing are only modestly correlated with objective ones (e.g., reward processing; Kujawa and Burkhouse, 2017). Likewise, poor agreements between retrospective and prospective measures of CA exist (Baldwin et al., 2019). Given that these measures may reflect distinct facets of these variables, future studies using multiple measures may prove useful.

Nevertheless, while study results should be interpreted in light of these limitations, this work has several important theoretical and clinical contributions too. First, it extends the literature examining association between CA and depression and identifies two variables that may have implications for it: reward and threat processing (Eshel and Roiser, 2010; Liuzzi et al., 2024). By investigating associations between variables, the study not only highlights the essential implications of CA in clinically depressed inpatients, but singles out threat processing. Second, it acknowledges childhood SES when investigating these associations. By adopting this approach, the study suggests that the observed patterns may be robust. Finally, it seeks to advance current knowledge by investigating potential mechanisms that follow CA and contribute to depression. While its findings did not find support for the potential mediating role of reward processing or threat processing, the study underscores the relevance of research in this area and points out future directions.

## Data Availability

The raw data supporting the conclusions of this article will be made available by the authors, without undue reservation.
